# Supplementary material for: Proteomic Analysis of Methylglyoxal Modifications Reveals Susceptibility of Glycolytic Enzymes to Dicarbonyl Stress
Source: Int J Mol Sci. 2022 Mar 28;23(7):3689. doi: 10.3390/ijms23073689 (PMC8998448; doi:10.3390/ijms23073689)
Supplement: Supplementary file 1 [file ijms-23-03689-s001.zip › ijms-1621919-Supplementary/Supplementary S1. Supplementary Figures 1_2 and supplementary table 1_2.pdf]

Supplementary Table S1

Protein	Gene	Count	MG-H (R) position	CEA (R) position	CEL (K) position
Actin, cytoplasmic 1	ACTB	19	28, 37, 39, 95, 206, 254, 256, 336	28, 37, 95, 116,	50, 61, 113, 238, 326, 328, 359
POTE ankyrin domain family member F	POTEF	9	728, 737, 954, 956	728, 737, 816	813, 938
Histone H2B	H2B	9	34, 80, 93	80, 93	109, 117, 121, 126
Peptidyl-prolyl cis-trans isomerase A	PPIA	7	55, 69 144	55, 69, 144	82
Tubulin alpha-1C chain	TUBA1C	6	84	84, 308, 390, 422	304
Alpha-enolase	ENO1	7	56	253	5, 54, 71, 193, 228

Top 6 proteins containing highest number of MGO modifications.

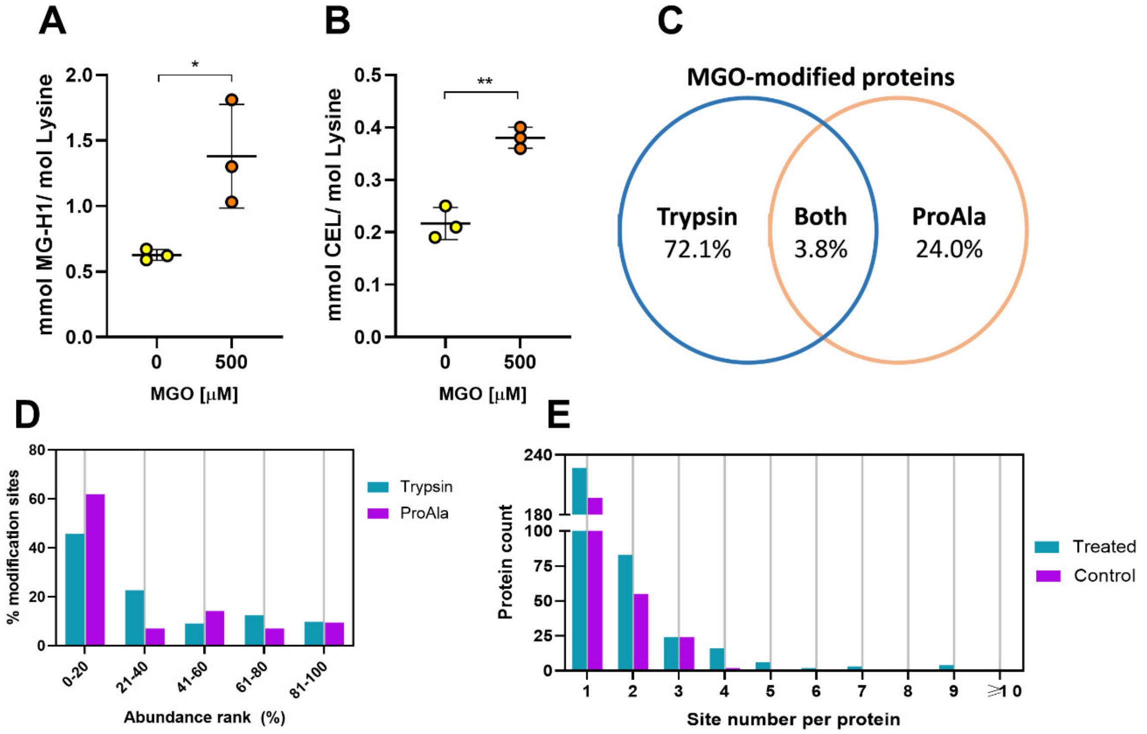

Supplementary Figure S1. A. MG-H1 concentration in control and cells treated with 500 µmol/L MGO for 24 hours. Mean ± SD, n=3, *p<0.05. **B**. CEL concentration in control and cells treated with 500 µmol/L MGO for 24 hours. Mean ± SD, n=3, ** p<0.01. **C**. Venn diagram showing the percentage of modified proteins detected in trypsin digests, ProAla digests or both (http://bioinformatics.psb.ugent.be/software/details/Venn-Diagrams). **D**. Percent of modification sites occurring on proteins based on protein rank. **E.** Number of modification sites per protein.

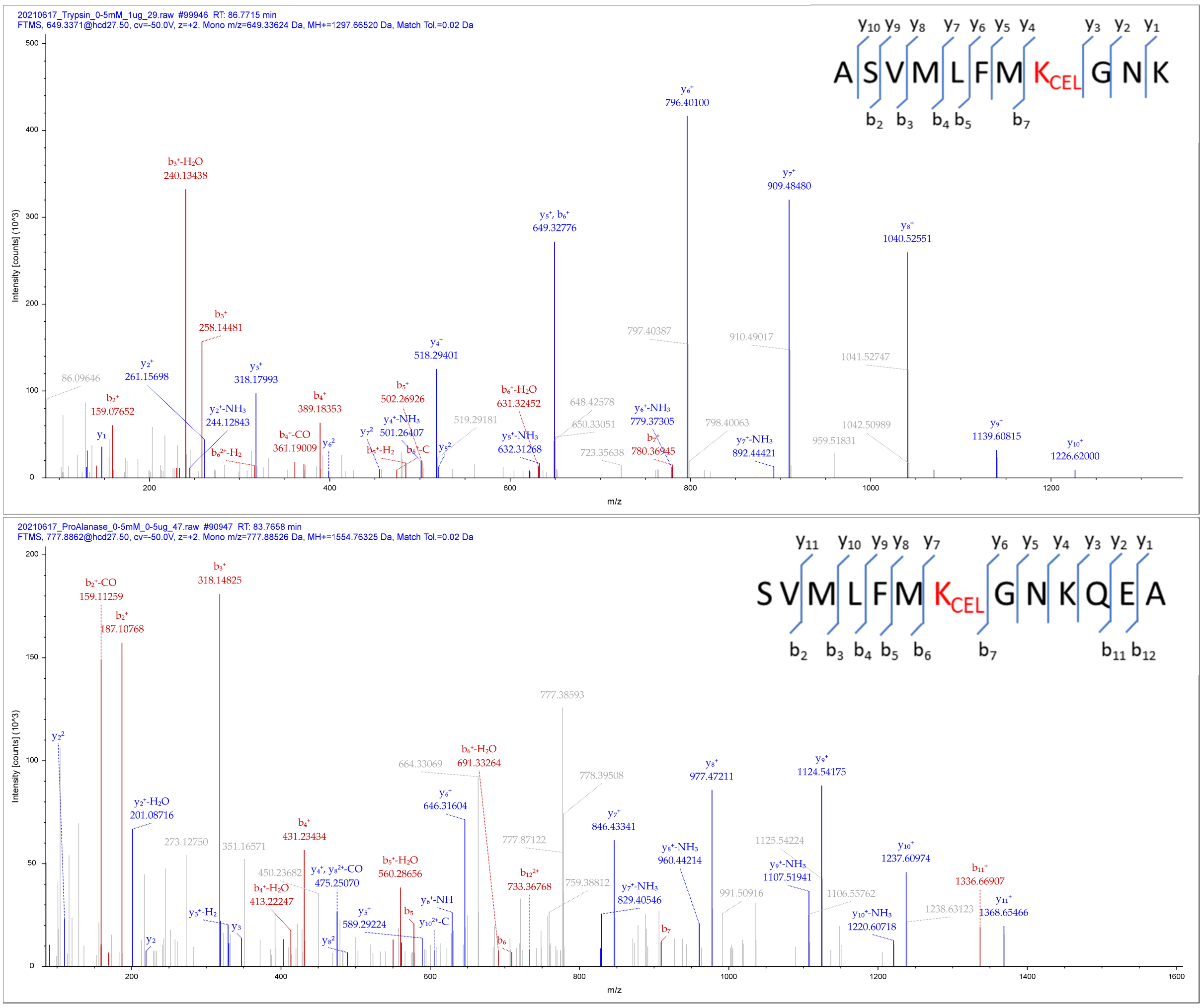

Supplementary Figure S2. Unedited MS/MS spectra of a CEL modification of Glutaredoxin at K253. Top; peptide generated in tryptic digest. Bottom; peptide generated in ProAla digest.

Supplementary Table S2

Accession	Protein	Peptide	Site	OV90 (Parental)	OV90 (CBPR)	Caov3 (Parental)	Caov3 (CBPR)	WIL2-NS	PBL
Q02539	Histone H1.1	E$\mathbf{R}_{\mathrm{CEA}}$GGVSLAALK<br>E$\mathbf{R}_{\mathrm{CEA}}$GGVSLAALKK	R57	+	+	+	+	+	+
F8WCU9	AT-rich interactive domain-containing protein 2 (Fragment)	DLISD$\mathbf{R}_{\mathrm{CEA}}$NK	R277	+	+	+	+	+	-
Q09666	Neuroblast differentiation-associated protein AHNAK	ISMPDVDLNL$\mathbf{K}_{\mathrm{CEL}}$GPK	K1333	+	+	+	-	+	+
J3KN16	Proteasome adapter and scaffold protein ECM29	MY$\mathbf{K}_{\mathrm{CEL}}$VYLGDIPLK	K476	+	+	+	+	-	-
Q7Z6Z7	E3 ubiquitin-protein ligase HUWE1	AALG$\mathbf{R}_{\mathrm{MG-H}}$ALAMAESTEK	R2162	+	+	+	+	-	-
Q9BXW9	Fanconi anemia group D2 protein	LVSPLC*LAPYF$\mathbf{R}_{\mathrm{CEA}}$LLR	R735	+	+	+	+	-	-
Q9Y490	Talin-1	ALSTDPAAPNL$\mathbf{K}_{\mathrm{CEL}}$SQLAAAA$\mathbf{R}_{\mathrm{MG-H}}$	K1332, R1340	+	+	+	+	-	-
Q16222	UDP-N-acetylhexosamine pyrophosphorylase	KENVIFFQQGMLPAMSFDG$\mathbf{K}_{\mathrm{CEL}}$IILEEK	K188	+	+	+	+	-	-
P04075	Fructose-bisphosphate aldolase A	ALSDHHIYLEGTLL$\mathbf{K}_{\mathrm{CEL}}$PNMVTPGHACTQK	K229	-	+	+	+	+	-
P08238	Heat shock protein HSP 90-beta	ADHGEPIG$\mathbf{R}_{\mathrm{MG-H}}GT\mathbf{K}_{\mathrm{CEL}}$VILHLKEDQTEYLEER	R177, K180	+	+	+	-	+	-

Protein residues containing MGO modifications that were observed in at least 4 of the 6-cell population. Untreated WIL2-NS were used for this analysis. (+) identified. (-) not identified. *Carbamidomethyl
