# Supplementary material for: Proteomic Analysis of Methylglyoxal Modifications Reveals Susceptibility of Glycolytic Enzymes to Dicarbonyl Stress
Source: Int J Mol Sci. 2022 Mar 28;23(7):3689. doi: 10.3390/ijms23073689 (PMC8998448; doi:10.3390/ijms23073689)

Supplementary S3. MS/MS spectra of all modifications identified on glycolytic enzymes

Fructose bisphosphate aldolase A (K147)

Sequence: DGADFAKWR, K7-Carboxyethyl (72.02113 Da)

Charge: +2, Monoisotopic m/z: 569.26703 Da (-2.78 mmu/-4.88 ppm), MH+: 1137.52679 Da, RT: 73.5025 min,

Identified with: Sequest HT (v1.17); **XCorr:3.04**, Percolator q-Value:1.9e-3, Percolator PEP:2.4e-2,

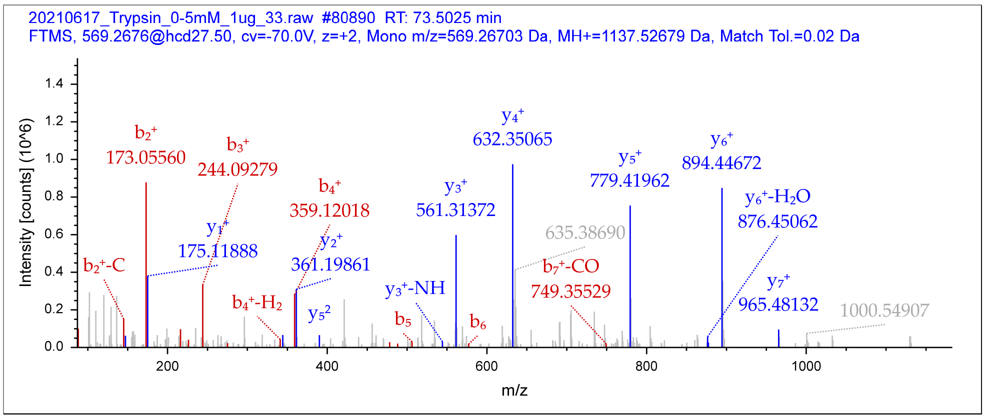

#1	b⁺	b²⁺	Seq.	y⁺	y²⁺	#2
1	116.03422	58.52075	D			9
2	173.05568	87.03148	G	1022.505...	511.75634	8
3	244.09280	122.55004	A	965.48394	483.24561	7
4	359.11974	180.06351	D	894.44683	447.72705	6
5	506.18815	253.59772	F	779.41988	390.21358	5
6	577.22527	289.11627	A	632.35147	316.67937	4
7	777.34136	389.17432	K-Carb...	561.31436	281.16082	3
8	963.42067	482.21397	W	361.19827	181.10277	2
9			R	175.11895	88.06311	1

Fructose bisphosphate aldolase A R42 (uniprot R43)

Sequence: RLQSIGTENTEENRR, R1-Carboxyethyl (72.02113 Da)

Charge: +3,   Monoisotopic m/z: 625.65026 Da (+1.58 mmu/+2.53 ppm),   MH+: 1874.93623 Da,   RT: 42.2328 min,

Identified with: Sequest HT (v1.17); **XCorr:1.82**, Percolator q-Value:3.8e-4, Percolator PEP:3.2e-3,

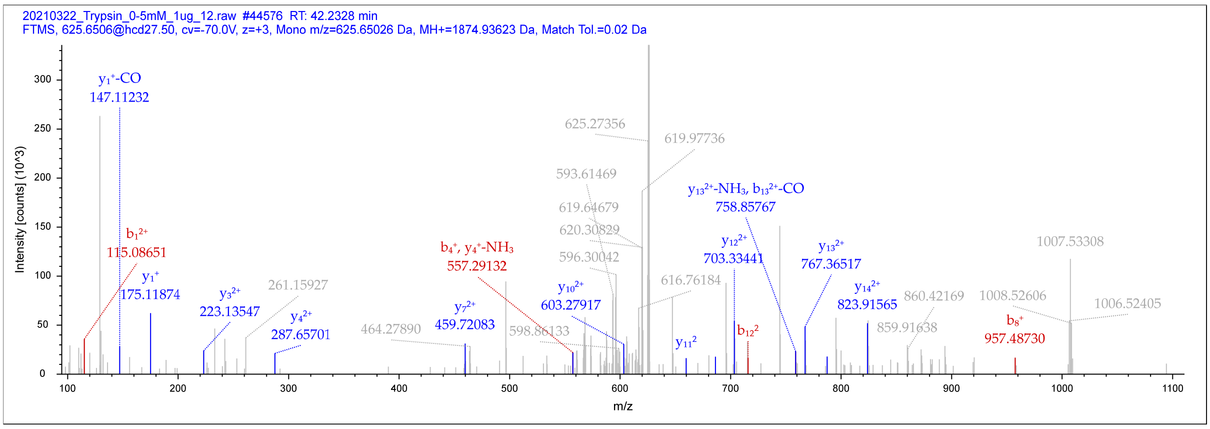

#1	b⁺	b²⁺	b³⁺	Seq.	y⁺	y²⁺	y³⁺	#2
1	229.12952	115.06840	77.04802	R-Car...				15
2	342.21358	171.61043	114.74271	L	1646.809...	823.90826	549.60793	14
3	470.27216	235.63972	157.42890	Q	1533.725...	767.36623	511.91325	13
4	557.30419	279.15573	186.43958	S	1405.666...	703.33694	469.22705	12
5	670.38825	335.69776	224.13427	I	1318.634...	659.82093	440.21638	11
6	727.40971	364.20850	243.14142	G	1205.550...	603.27890	402.52169	10
7	828.45739	414.73233	276.82398	T	1148.529...	574.76817	383.51454	9
8	957.49999	479.25363	319.83818	E	1047.481...	524.24433	349.83198	8
9	1071.542...	536.27509	357.85249	N	918.43878	459.72303	306.81778	7
10	1172.590...	586.79893	391.53505	T	804.39586	402.70157	268.80347	6
11	1301.633...	651.32023	434.54925	E	703.34818	352.17773	235.12091	5
12	1430.675...	715.84153	477.56344	E	574.30558	287.65643	192.10671	4
13	1544.718...	772.86299	515.57775	N	445.26299	223.13513	149.09251	3
14	1700.819...	850.91355	567.61146	R	331.22006	166.11367	111.07821	2
15				R	175.11895	88.06311	59.04450	1

Fructose bisphosphate aldolase K229 (uniprot K230)

Sequence: ALSDHHIYLEGTLLKPNMVTPGHACTQK, C25-Carbamidomethyl (57.02146 Da), K15-Carboxyethyl (72.02113 Da)

Charge: +5, Monoisotopic m/z: 641.52969 Da (+4.35 mmu/+6.78 ppm), MH+: 3203.61933 Da, RT: 82.2256 min,

Identified with: Sequest HT (v1.17); **XCorr:3.97**, Percolator q-Value:9.6e-5, Percolator PEP:4.9e-4,

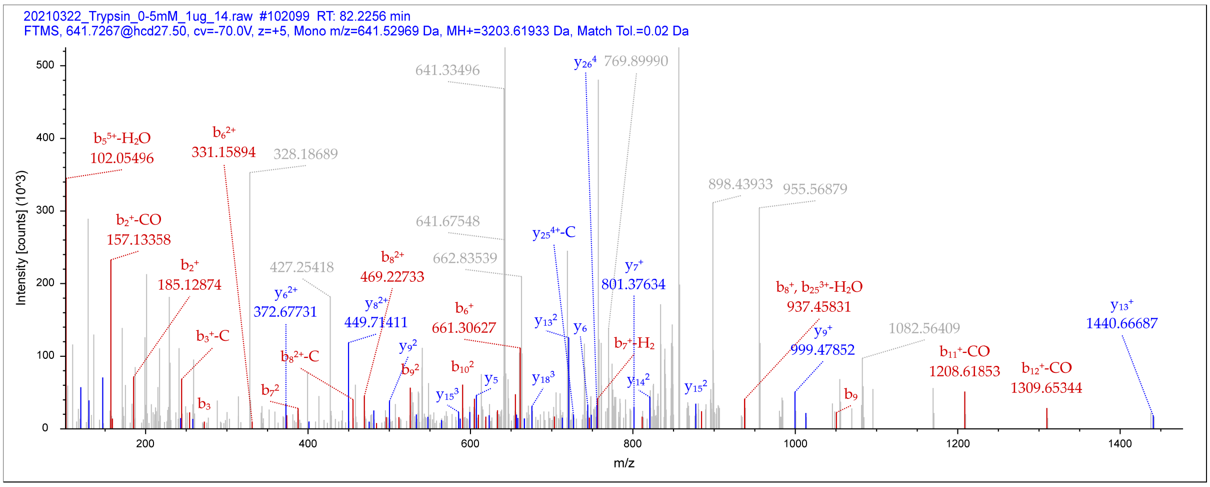

#1	b⁺	b²⁺	b³⁺	b⁴⁺	b⁵⁺	Seq.	y⁺	y²⁺	y³⁺	y⁴⁺	y⁵⁺	#2
1	72.04439	36.52583	24.68631	18.76656	15.21470	A						28
2	185.12845	93.06787	62.38100	47.03757	37.83151	L	3132.560...	1566.783...	1044.858...	783.89558	627.31792	27
3	272.16048	136.58388	91.39168	68.79558	55.23792	S	3019.476...	1510.241...	1007.163...	755.62457	604.70111	26
4	387.18743	194.09735	129.73399	97.55231	78.24331	D	2932.444...	1466.725...	978.15299	733.86656	587.29470	25
5	524.24634	262.62681	175.42030	131.81704	105.65509	H	2817.417...	1409.212...	939.81067	705.10982	564.28931	24
6	661.30525	331.15626	221.10660	166.08177	133.06687	H	2680.358...	1340.682...	894.12437	670.84510	536.87753	23
7	774.38931	387.69829	258.80129	194.35279	155.68368	I	2543.299...	1272.153...	848.43806	636.58037	509.46575	22
8	937.45264	469.22996	313.15573	235.11862	188.29635	Y	2430.215...	1215.611...	810.74338	608.30935	486.84894	21
9	1050.536...	525.77199	350.85042	263.38963	210.91316	L	2267.152...	1134.079...	756.38893	567.54352	454.23627	20
10	1179.579...	590.29329	393.86462	295.65028	236.72168	E	2154.068...	1077.537...	718.69425	539.27250	431.61946	19
11	1236.600...	618.80402	412.87177	309.90565	248.12597	G	2025.025...	1013.016...	675.68005	507.01186	405.81094	18
12	1337.648...	669.32786	446.55433	335.16757	268.33551	T	1968.004...	984.50570	656.67289	492.75649	394.40665	17
13	1450.732...	725.86989	484.24902	363.43858	290.95232	L	1866.956...	933.98186	622.99033	467.49457	374.19711	16
14	1563.816...	782.41192	521.94371	391.70960	313.56914	L	1753.872...	877.43983	585.29565	439.22355	351.58030	15
15	1763.932...	882.46997	588.64907	441.73862	353.59235	K-Carb...	1640.788...	820.89780	547.60096	410.95254	328.96349	14
16	1860.985...	930.99635	620.99999	466.00181	373.00291	P	1440.672...	720.83975	480.89559	360.92351	288.94027	13
17	1975.028...	988.01781	659.01430	494.51255	395.81149	N	1343.619...	672.31337	448.54467	336.66032	269.52971	12
18	2106.068...	1053.538...	702.69446	527.27267	422.01959	M	1229.576...	615.29191	410.53036	308.14959	246.72113	11
19	2205.137...	1103.072...	735.71727	552.03977	441.83327	V	1098.536...	549.77167	366.85020	275.38947	220.51303	10
20	2306.184...	1153.596...	769.39983	577.30169	462.04281	T	999.46764	500.23746	333.82740	250.62237	200.69935	9
21	2403.237...	1202.122...	801.75075	601.56488	481.45336	P	898.41996	449.71362	300.14484	225.36045	180.48981	8
22	2460.259...	1230.633...	820.75790	615.82025	492.85765	G	801.36720	401.18724	267.79392	201.09726	161.07926	7
23	2597.318...	1299.162...	866.44421	650.08497	520.26943	H	744.34573	372.67651	248.78676	186.84189	149.67497	6
24	2668.355...	1334.681...	890.12324	667.84425	534.47686	A	607.28682	304.14705	203.10046	152.57716	122.26319	5
25	2828.385...	1414.696...	943.46679	707.85191	566.48299	C-Car...	536.24971	268.62849	179.42142	134.81788	108.05576	4
26	2929.433...	1465.220...	977.14935	733.11383	586.69252	T	376.21906	188.61317	126.07787	94.81022	76.04963	3
27	3057.492...	1529.249...	1019.835...	765.12848	612.30424	Q	275.17138	138.08933	92.39531	69.54830	55.84010	2
28						K	147.11280	74.06004	49.70912	37.53366	30.22838	1

Fructose bisphosphate aldolase A K41 (uniprot K42)

Sequence: GILAADESTGSIAKR, K14-Carboxyethyl (72.02113 Da)

Charge: +2,   Monoisotopic m/z: 780.91548 Da (+0.46 mmu/+0.59 ppm),   MH+: 1560.82369 Da,   RT: 67.7994 min,

Identified with: Sequest HT (v1.17); **XCorr:1.18**, Percolator q-Value:4.9e-5, Percolator PEP:2.7e-4,

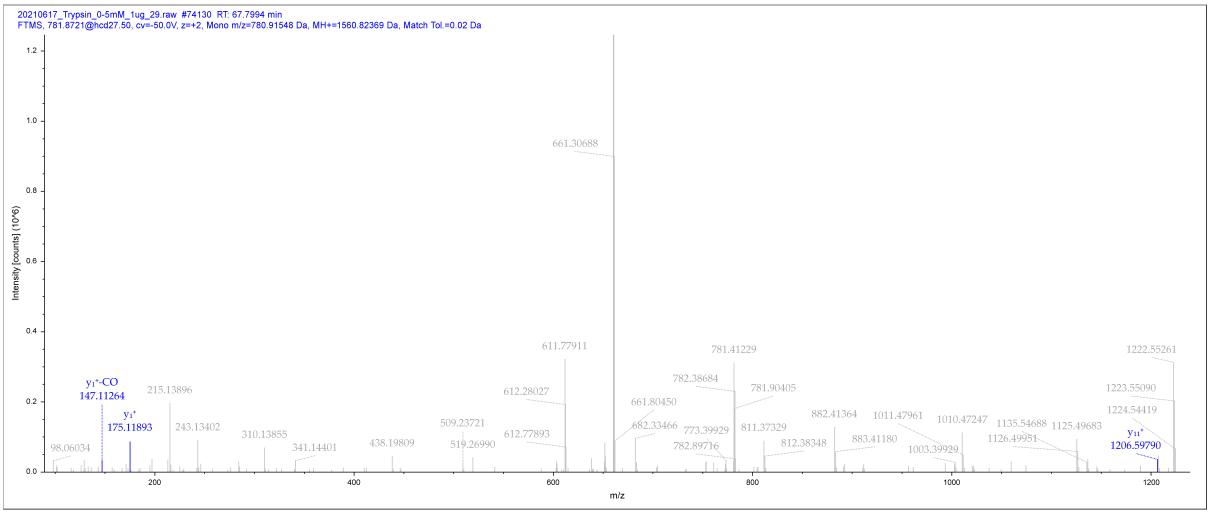

#1	b⁺	b²⁺	Seq.	y⁺	y²⁺	#2
1	58.02874	29.51801	G			15
2	171.11280	86.06004	I	1503.801...	752.40429	14
3	284.19687	142.60207	L	1390.717...	695.86226	13
4	355.23398	178.12063	A	1277.633...	639.32023	12
5	426.27110	213.63919	A	1206.596...	603.80167	11
6	541.29804	271.15266	D	1135.558...	568.28312	10
7	670.34063	335.67395	E	1020.532...	510.76965	9
8	757.37266	379.18997	S	891.48942	446.24835	8
9	858.42034	429.71381	T	804.45739	402.73233	7
10	915.44180	458.22454	G	703.40971	352.20850	6
11	1002.473...	501.74055	S	646.38825	323.69776	5
12	1115.557...	558.28259	I	559.35622	280.18175	4
13	1186.595...	593.80114	A	446.27216	223.63972	3
14	1386.711...	693.85919	K-Carb...	375.23504	188.12116	2
15			R	175.11895	88.06311	1

Alpha enolase (R253)

Sequence: SEFFRSGKYDLDFKSPDDPSRYISP, R5-Carboxyethyl (72.02113 Da)

Charge: +3, Monoisotopic m/z: 1009.15263 Da (+7.31 mmu/+7.25 ppm), MH+: 3025.44333 Da, RT: 96.1008 min,

Identified with: Sequest HT (v1.17); **XCorr:2.19**, Percolator q-Value:1.6e-3, Percolator PEP:6.0e-3,

#1	b^+	b^{2+}	b^{3+}	Seq.	y^+	y^{2+}	y^{3+}	#2
1	88.03930	44.52329	30.01795	S				25
2	217.08190	109.04459	73.03215	E	2938.389...	1469.698...	980.13464	24
3	364.15031	182.57879	122.05496	F	2809.346...	1405.177...	937.12045	23
4	511.21873	256.11300	171.07776	F	2662.278...	1331.642...	888.09764	22
5	739.34097	370.17412	247.11851	R-Car...	2515.209...	1258.108...	839.07484	21
6	826.37299	413.69014	276.12918	S	2287.087...	1144.047...	763.03409	20
7	883.39446	442.20087	295.13634	G	2200.055...	1100.531...	734.02341	19
8	1011.489...	506.24835	337.83466	K	2143.034...	1072.020...	715.01626	18
9	1174.552...	587.78001	392.18910	Y	2014.939...	1007.973...	672.31794	17
10	1289.579...	645.29348	430.53142	D	1851.875...	926.44161	617.96350	16
11	1402.663...	701.83552	468.22610	L	1736.848...	868.92813	579.62118	15
12	1517.690...	759.34899	506.56842	D	1623.764...	812.38610	541.92649	14
13	1664.759...	832.88319	555.59122	F	1508.737...	754.87263	503.58418	13
14	1792.854...	896.93068	598.28954	K	1361.669...	681.33842	454.56137	12
15	1879.886...	940.44669	627.30022	S	1233.574...	617.29094	411.86305	11
16	1976.938...	988.97307	659.65114	P	1146.542...	573.77493	382.85238	10
17	2091.965...	1046.486...	697.99345	D	1049.489...	525.24855	350.50146	9
18	2206.992...	1104.000...	736.33577	D	934.46287	467.73507	312.15914	8
19	2304.045...	1152.526...	768.68669	P	819.43593	410.22160	273.81683	7
20	2391.077...	1196.042...	797.69737	S	722.38317	361.69522	241.46591	6
21	2547.178...	1274.092...	849.73107	R	635.35114	318.17921	212.45523	5
22	2710.241...	1355.624...	904.08551	Y	479.25003	240.12865	160.42153	4
23	2823.326...	1412.166...	941.78020	I	316.18670	158.59699	106.06708	3
24	2910.358...	1455.682...	970.79088	S	203.10263	102.05496	68.37240	2
25				P	116.07061	58.53894	39.36172	1

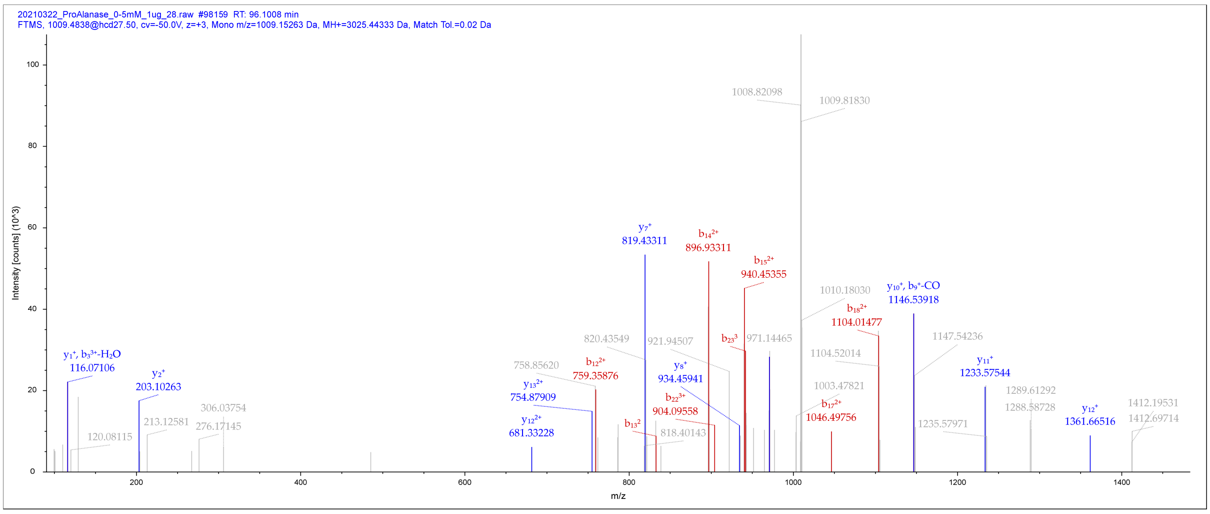

Alpha enolase (R56)

Sequence: AAVPSGASTGIYEALELRDNDKTRYMGK, R24-MG-H1 (54.01057 Da)

Charge: +5,   Monoisotopic m/z: 614.30875 Da (-0.14 mmu/-0.23 ppm),   MH+: 3067.51463 Da,   RT: 82.8454 min,

Identified with: Sequest HT (v1.17); XCorr:4.77, Percolator q-Value:2.6e-5, Percolator PEP:8.3e-5,

#1	b^+	b^{2+}	b^{3+}	b^{4+}	b^{5+}	Seq.	y^+	y^{2+}	y^{3+}	y^{4+}	y^{5+}	#2
1	72.04439	36.52583	24.68631	18.76656	15.21470	A						28
2	143.08150	72.04439	48.36535	36.52583	29.42212	A	2996.478...	1498.742...	999.49759	749.87501	600.10146	27
3	242.14992	121.57860	81.38816	61.29294	49.23580	V	2925.441...	1463.224...	975.81855	732.11573	585.89404	26
4	339.20268	170.10498	113.73908	85.55613	68.64636	P	2826.372...	1413.689...	942.79575	707.34863	566.08036	25
5	426.23471	213.62099	142.74975	107.31414	86.05276	S	2729.319...	1365.163...	910.44482	683.08544	546.66981	24
6	483.25617	242.13173	161.75691	121.56950	97.45706	G	2642.287...	1321.647...	881.43415	661.32743	529.26340	23
7	554.29329	277.65028	185.43595	139.32878	111.66448	A	2585.266...	1293.136...	862.42699	647.07206	517.85911	22
8	641.32532	321.16630	214.44662	161.08679	129.07088	S	2514.229...	1257.618...	838.74796	629.31279	503.65168	21
9	742.37299	371.69014	248.12918	186.34871	149.28042	T	2427.197...	1214.102...	809.73728	607.55478	486.24528	20
10	799.39446	400.20087	267.13634	200.60407	160.68471	G	2326.149...	1163.578...	776.05472	582.29286	466.03574	19
11	912.47852	456.74290	304.83103	228.87509	183.30153	I	2269.128...	1135.067...	757.04757	568.03749	454.63145	18
12	1075.541...	538.27456	359.18547	269.64092	215.91419	Y	2156.044...	1078.525...	719.35288	539.76648	432.01464	17
13	1204.584...	602.79586	402.19967	301.90157	241.72271	E	1992.980...	996.99401	664.99843	499.00065	399.40197	16
14	1275.621...	638.31442	425.87870	319.66085	255.93013	A	1863.938...	932.47272	621.98424	466.74000	373.59345	15
15	1388.705...	694.85645	463.57339	347.93186	278.54695	L	1792.901...	896.95416	598.30520	448.98072	359.38603	14
16	1517.748...	759.37775	506.58759	380.19251	304.35546	E	1679.816...	840.41213	560.61051	420.70970	336.76922	13
17	1630.832...	815.91978	544.28228	408.46353	326.97228	L	1550.774...	775.89083	517.59631	388.44905	310.96070	12
18	1786.933...	893.97033	596.31598	447.48880	358.19250	R	1437.690...	719.34880	479.90163	360.17804	288.34389	11
19	1901.960...	951.48380	634.65830	476.24554	381.19789	D	1281.589...	641.29824	427.86792	321.15276	257.12366	10
20	2016.003...	1008.505...	672.67260	504.75627	404.00647	N	1166.562...	583.78477	389.52561	292.39602	234.11828	9
21	2131.030...	1066.018...	711.01492	533.51301	427.01186	D	1052.519...	526.76331	351.51130	263.88529	211.30969	8
22	2259.125...	1130.066...	753.71324	565.53675	452.63085	K	937.49240	469.24984	313.16898	235.12856	188.30430	7
23	2360.172...	1180.590...	787.39580	590.79867	472.84039	T	809.39744	405.20236	270.47066	203.10482	162.68531	6
24	2570.284...	1285.645...	857.43302	643.32659	514.86273	R-MG-...	708.34976	354.67852	236.78810	177.84290	142.47577	5
25	2733.347...	1367.177...	911.78747	684.09242	547.47539	Y	498.23808	249.62268	166.75088	125.31498	100.45344	4
26	2864.388...	1432.697...	955.46763	716.85254	573.68349	M	335.17475	168.09101	112.39644	84.54915	67.84077	3
27	2921.409...	1461.208...	974.47478	731.10791	585.08778	G	204.13427	102.57077	68.71627	51.78902	41.63267	2
28						K	147.11280	74.06004	49.70912	37.53366	30.22838	1

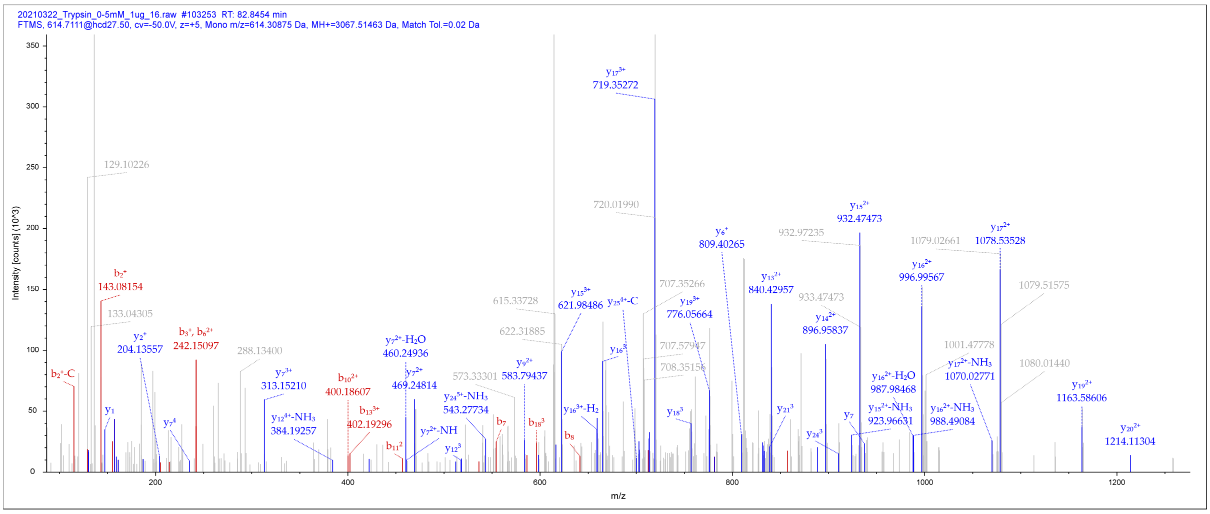

Alpha enolase (K71)

Sequence: AVEHINKTIAPALVSK, K7-Carboxyethyl (72.02113 Da)

Charge: +3, Monoisotopic m/z: 588.34208 Da (+1.84 mmu/+3.13 ppm), MH+: 1763.01167 Da, RT: 72.0724 min,

Identified with: Sequest HT (v1.17); **XCorr:3.57**, Percolator q-Value:1.0e-4, Percolator PEP:6.5e-4,

#1	b+	b2+	b3+	Seq.	y+	y2+	y3+	#2
1	72.04439	36.52583	24.68631	A				16
2	171.11280	86.06004	57.70912	V	1691.969...	846.48816	564.66120	15
3	300.15540	150.58134	100.72332	E	1592.900...	796.95395	531.63840	14
4	437.21431	219.11079	146.40962	H	1463.858...	732.43266	488.62420	13
5	550.29837	275.65282	184.10431	I	1326.799...	663.90320	442.93789	12
6	664.34130	332.67429	222.11862	N	1213.715...	607.36117	405.24321	11
7	864.45739	432.73233	288.82398	K-Carb...	1099.672...	550.33971	367.22890	10
8	965.50507	483.25617	322.50654	T	899.55604	450.28166	300.52353	9
9	1078.589...	539.79821	360.20123	I	798.50837	399.75782	266.84097	8
10	1149.626...	575.31676	383.88027	A	685.42430	343.21579	229.14628	7
11	1246.679...	623.84314	416.23119	P	614.38719	307.69723	205.46725	6
12	1317.716...	659.36170	439.91023	A	517.33442	259.17085	173.11633	5
13	1430.800...	715.90373	477.60491	L	446.29731	223.65229	149.43729	4
14	1529.868...	765.43794	510.62772	V	333.21325	167.11026	111.74260	3
15	1616.900...	808.95395	539.63840	S	234.14483	117.57605	78.71980	2
16				K	147.11280	74.06004	49.70912	1

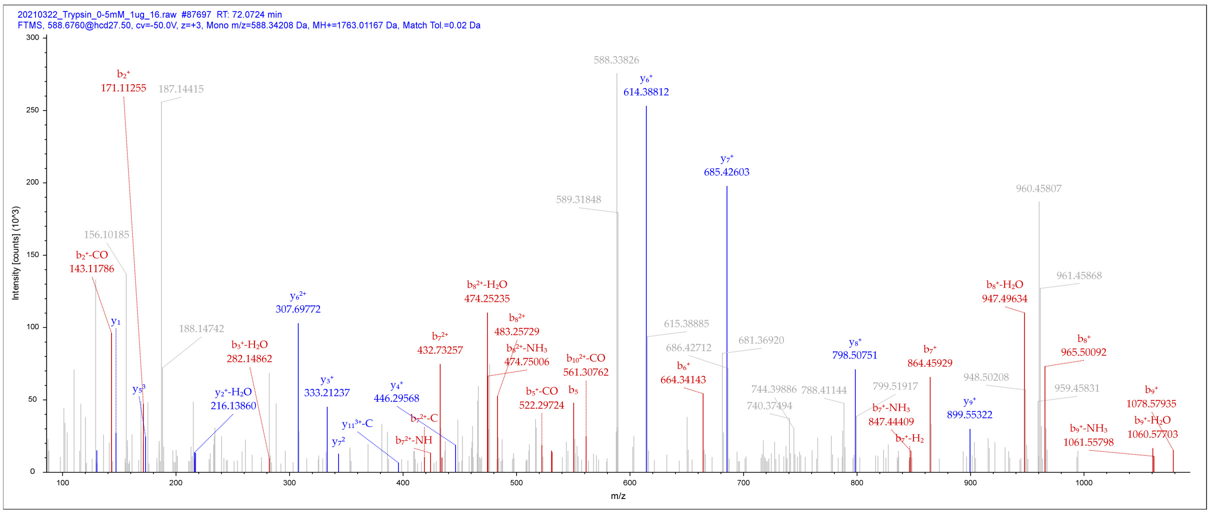

Alpha enolase (K54)

Sequence: AAVPSGASTGIYEALELRDNDKTR, K22-Carboxyethyl (72.02113 Da)

Charge: +3, Monoisotopic m/z: 869.43987 Da (-0.2 mmu/-0.23 ppm), MH+: 2606.30505 Da, RT: 90.7175 min,

Identified with: Sequest HT (v1.17); **XCorr:2.53**, Percolator q-Value:4.9e-5, Percolator PEP:2.8e-4,

#1	b^+	b^{2+}	b^{3+}	Seq.	y^+	y^{2+}	y^{3+}	#2
1	72.04439	36.52583	24.68631	A				24
2	143.08150	72.04439	48.36535	A	2535.268...	1268.137...	845.76103	23
3	242.14992	121.57860	81.38816	V	2464.231...	1232.619...	822.08199	22
4	339.20268	170.10498	113.73908	P	2365.163...	1183.085...	789.05919	21
5	426.23471	213.62099	142.74975	S	2268.110...	1134.558...	756.70827	20
6	483.25617	242.13173	161.75691	G	2181.078...	1091.042...	727.69759	19
7	554.29329	277.65028	185.43595	A	2124.056...	1062.532...	708.69043	18
8	641.32532	321.16630	214.44662	S	2053.019...	1027.013...	685.01140	17
9	742.37299	371.69014	248.12918	T	1965.987...	983.49744	656.00072	16
10	799.39446	400.20087	267.13634	G	1864.939...	932.97360	622.31816	15
11	912.47852	456.74290	304.83103	I	1807.918...	904.46287	603.31101	14
12	1075.541...	538.27456	359.18547	Y	1694.834...	847.92084	565.61632	13
13	1204.584...	602.79586	402.19967	E	1531.771...	766.38918	511.26188	12
14	1275.621...	638.31442	425.87870	A	1402.728...	701.86788	468.24768	11
15	1388.705...	694.85645	463.57339	L	1331.691...	666.34932	444.56864	10
16	1517.748...	759.37775	506.58759	E	1218.607...	609.80729	406.87395	9
17	1630.832...	815.91978	544.28228	L	1089.564...	545.28599	363.85975	8
18	1786.933...	893.97033	596.31598	R	976.48065	488.74396	326.16507	7
19	1901.960...	951.48380	634.65830	D	820.37954	410.69341	274.13136	6
20	2016.003...	1008.505...	672.67260	N	705.35259	353.17993	235.78905	5
21	2131.030...	1066.018...	711.01492	D	591.30967	296.15847	197.77474	4
22	2331.146...	1166.076...	777.72028	K-Carb...	476.28272	238.64500	159.43243	3
23	2432.193...	1216.600...	811.40284	T	276.16663	138.58695	92.72706	2
24				R	175.11895	88.06311	59.04450	1

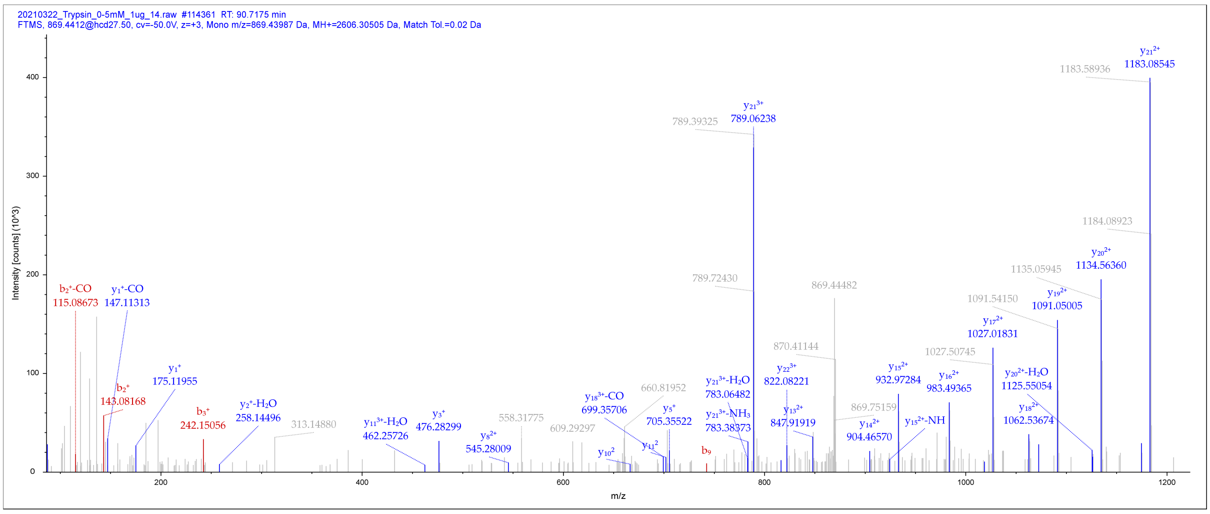

Alpha enolase (K193)

Sequence: IGAEVYHNLKNVIKEK, K10-Carboxyethyl (72.02113 Da)

Charge: +3,   Monoisotopic m/z: 643.02736 Da (+0.93 mmu/+1.45 ppm),   MH+: 1927.06753 Da,   RT: 79.8869 min,

Identified with: Sequest HT (v1.17); **XCorr:2.06**, Percolator q-Value:4.6e-5, Percolator PEP:2.2e-4,

#1	b^+	b^{2+}	b^{3+}	Seq.	y^+	y^{2+}	y^{3+}	#2
1	114.09134	57.54931	38.70196	I				16
2	171.11280	86.06004	57.70912	G	1813.980...	907.49397	605.33174	15
3	242.14992	121.57860	81.38816	A	1756.959...	878.98324	586.32459	14
4	371.19251	186.09989	124.40235	E	1685.922...	843.46469	562.64555	13
5	470.26092	235.63410	157.42516	V	1556.879...	778.94339	519.63135	12
6	633.32425	317.16576	211.77960	Y	1457.811...	729.40918	486.60855	11
7	770.38317	385.69522	257.46591	H	1294.747...	647.87752	432.25410	10
8	884.42609	442.71668	295.48022	N	1157.688...	579.34806	386.56780	9
9	997.51016	499.25872	333.17490	L	1043.645...	522.32660	348.55349	8
10	1197.626...	599.31676	399.88027	K-Carb...	930.56186	465.78457	310.85880	7
11	1311.669...	656.33823	437.89458	N	730.44577	365.72652	244.15344	6
12	1410.737...	705.87243	470.91738	V	616.40284	308.70506	206.13913	5
13	1523.821...	762.41447	508.61207	I	517.33442	259.17085	173.11633	4
14	1651.916...	826.46195	551.31039	K	404.25036	202.62882	135.42164	3
15	1780.959...	890.98324	594.32459	E	276.15540	138.58134	92.72332	2
16				K	147.11280	74.06004	49.70912	1

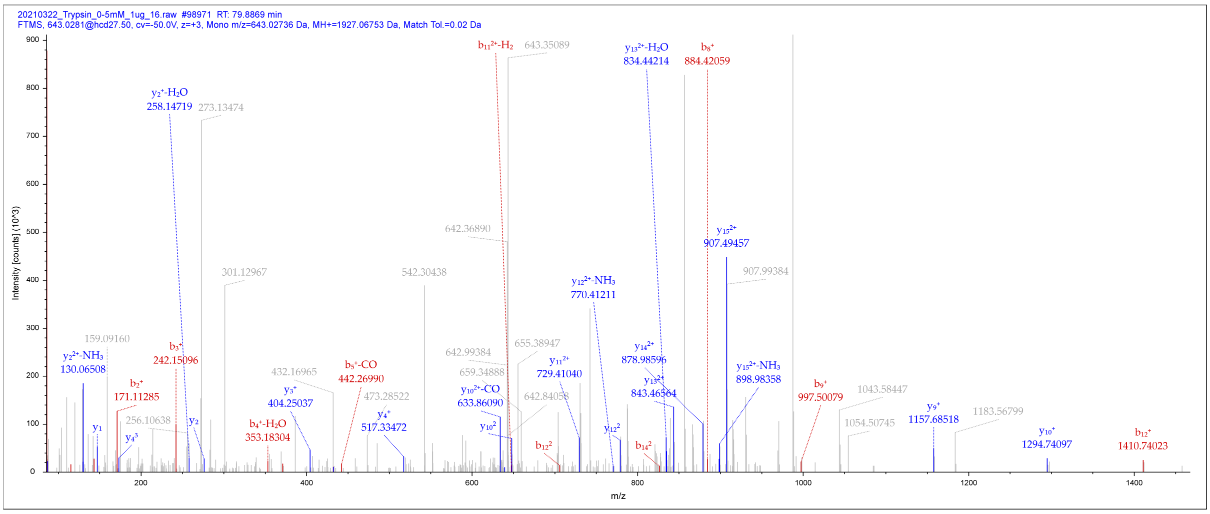

Alpha enolase (K5)

Sequence: MSILKIHAR, K5-Carboxyethyl (72.02113 Da), M1-Met-loss+Acetyl (-89.02992 Da)

Charge: +2, Monoisotopic m/z: 526.31662 Da (+0.05 mmu/+0.1 ppm), MH+: 1051.62596 Da, RT: 74.5176 min,

Identified with: Sequest HT (v1.17); **XCorr:2.76**, Percolator q-Value:2.0e-4, Percolator PEP:1.5e-3,

#1	b^+	b^{2+}	Seq.	y^+	y^{2+}	#2
1	43.01784	22.01256	M-Met-...			9
2	130.04987	65.52857	S	1009.615...	505.31128	8
3	243.13393	122.07061	I	922.58326	461.79527	7
4	356.21800	178.61264	L	809.49920	405.25324	6
5	556.33409	278.67068	K-Carb...	696.41513	348.71121	5
6	669.41815	335.21271	I	496.29904	248.65316	4
7	806.47707	403.74217	H	383.21498	192.11113	3
8	877.51418	439.26073	A	246.15607	123.58167	2
9			R	175.11895	88.06311	1

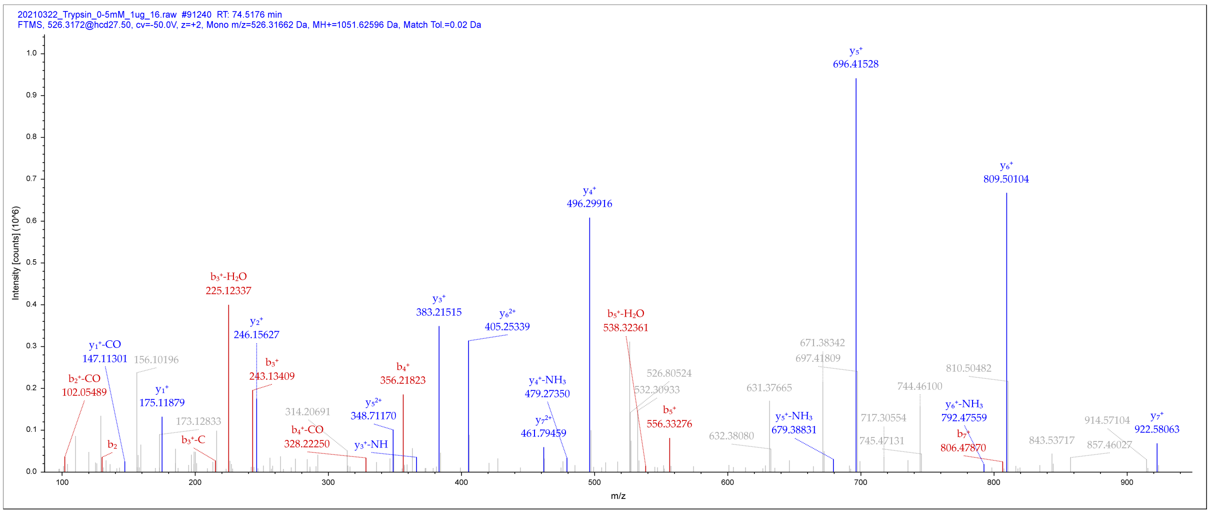

Beta enolase (R50)

Sequence: AAVPSGASTGIYEALELRDGDKGR, R18-Carboxyethyl (72.02113 Da)

Charge: +4, Monoisotopic m/z: 627.07520 Da (+5.26 mmu/+8.39 ppm), MH+: 2505.27898 Da, RT: 83.5223 min,

Identified with: Sequest HT (v1.17); **XCorr:2.37**, Percolator q-Value:4.3e-5, Percolator PEP:1.4e-4,

#1	b⁺	b²⁺	b³⁺	b⁴⁺	Seq.	y⁺	y²⁺	y³⁺	y⁴⁺	#2
1	72.04439	36.52583	24.68631	18.76656	A					24
2	143.08150	72.04439	48.36535	36.52583	A	2434.220...	1217.614...	812.07847	609.31067	23
3	242.14992	121.57860	81.38816	61.29294	V	2363.183...	1182.095...	788.39943	591.55139	22
4	339.20268	170.10498	113.73908	85.55613	P	2264.115...	1132.561...	755.37663	566.78429	21
5	426.23471	213.62099	142.74975	107.31414	S	2167.062...	1084.034...	723.02571	542.52110	20
6	483.25617	242.13173	161.75691	121.56950	G	2080.030...	1040.518...	694.01503	520.76309	19
7	554.29329	277.65028	185.43595	139.32878	A	2023.009...	1012.008...	675.00788	506.50773	18
8	641.32532	321.16630	214.44662	161.08679	S	1951.971...	976.48962	651.32884	488.74845	17
9	742.37299	371.69014	248.12918	186.34871	T	1864.939...	932.97360	622.31816	466.99044	16
10	799.39446	400.20087	267.13634	200.60407	G	1763.892...	882.44976	588.63560	441.72852	15
11	912.47852	456.74290	304.83103	228.87509	I	1706.870...	853.93903	569.62845	427.47315	14
12	1075.541...	538.27456	359.18547	269.64092	Y	1593.786...	797.39700	531.93376	399.20214	13
13	1204.584...	602.79586	402.19967	301.90157	E	1430.723...	715.86534	477.57932	358.43631	12
14	1275.621...	638.31442	425.87870	319.66085	A	1301.680...	651.34404	434.56512	326.17566	11
15	1388.705...	694.85645	463.57339	347.93186	L	1230.643...	615.82548	410.88608	308.41638	10
16	1517.748...	759.37775	506.58759	380.19251	E	1117.559...	559.28345	373.19139	280.14536	9
17	1630.832...	815.91978	544.28228	408.46353	L	988.51703	494.76215	330.17720	247.88472	8
18	1858.954...	929.98090	620.32302	465.49409	R-Car...	875.43297	438.22012	292.48251	219.61370	7
19	1973.981...	987.49437	658.66534	494.25082	D	647.31073	324.15900	216.44176	162.58314	6
20	2031.002...	1016.005...	677.67249	508.50619	G	532.28379	266.64553	178.09945	133.82640	5
21	2146.029...	1073.518...	716.01481	537.26292	D	475.26232	238.13480	159.09229	119.57104	4
22	2274.124...	1137.566...	758.71313	569.28667	K	360.23538	180.62133	120.74998	90.81430	3
23	2331.146...	1166.076...	777.72028	583.54203	G	232.14042	116.57385	78.05166	58.79056	2
24					R	175.11895	88.06311	59.04450	44.53520	1

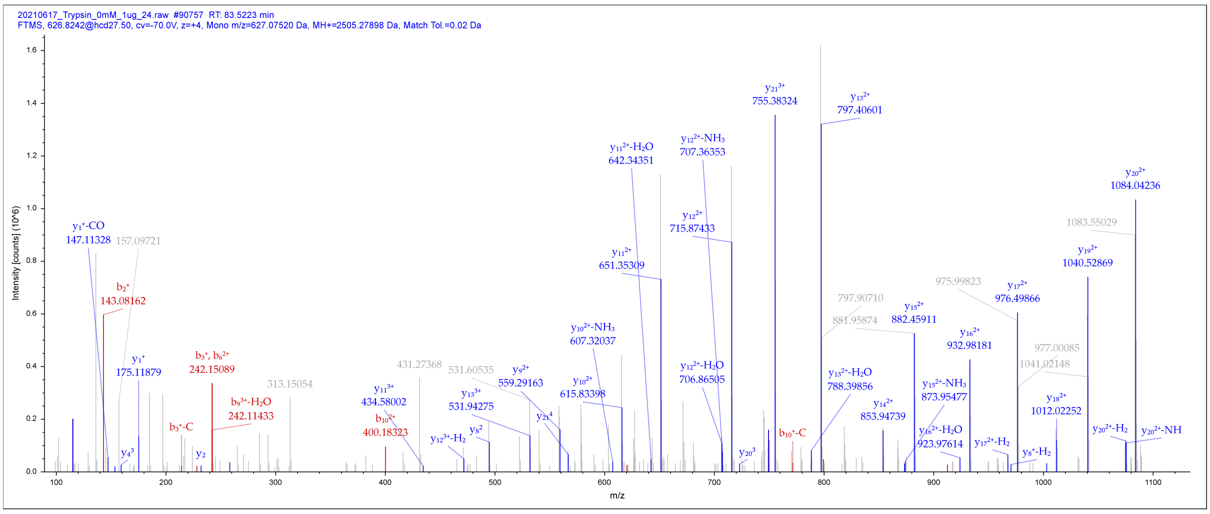

Glyceraldehyde-3-phosphate dehydrogenase (R234)

Sequence: ELNGKLTGMAFRVP, R12-MG-H1 (54.01057 Da)

Charge: +2,   Monoisotopic m/z: 793.92633 Da (+4.73 mmu/+5.95 ppm),   MH+: 1586.84537 Da,   RT: 93.3511 min,

Identified with: Sequest HT (v1.17); **XCorr:1.71**, Percolator q-Value:2.9e-3, Percolator PEP:1.2e-2,

#1	b^+	b^{2+}	Seq.	y^+	y^{2+}	#2
1	130.04987	65.52857	E			14
2	243.13393	122.07061	L	1457.793...	729.40030	13
3	357.17686	179.09207	N	1344.709...	672.85827	12
4	414.19832	207.60280	G	1230.666...	615.83681	11
5	542.29329	271.65028	K	1173.644...	587.32608	10
6	655.37735	328.19231	L	1045.549...	523.27859	9
7	756.42503	378.71615	T	932.46585	466.73656	8
8	813.44649	407.22689	G	831.41817	416.21272	7
9	944.48698	472.74713	M	774.39671	387.70199	6
10	1015.524...	508.26568	A	643.35622	322.18175	5
11	1162.592...	581.79989	F	572.31911	286.66319	4
12	1372.704...	686.85573	R-MG-...	425.25069	213.12899	3
13	1471.772...	736.38994	V	215.13902	108.07315	2
14			P	116.07061	58.53894	1

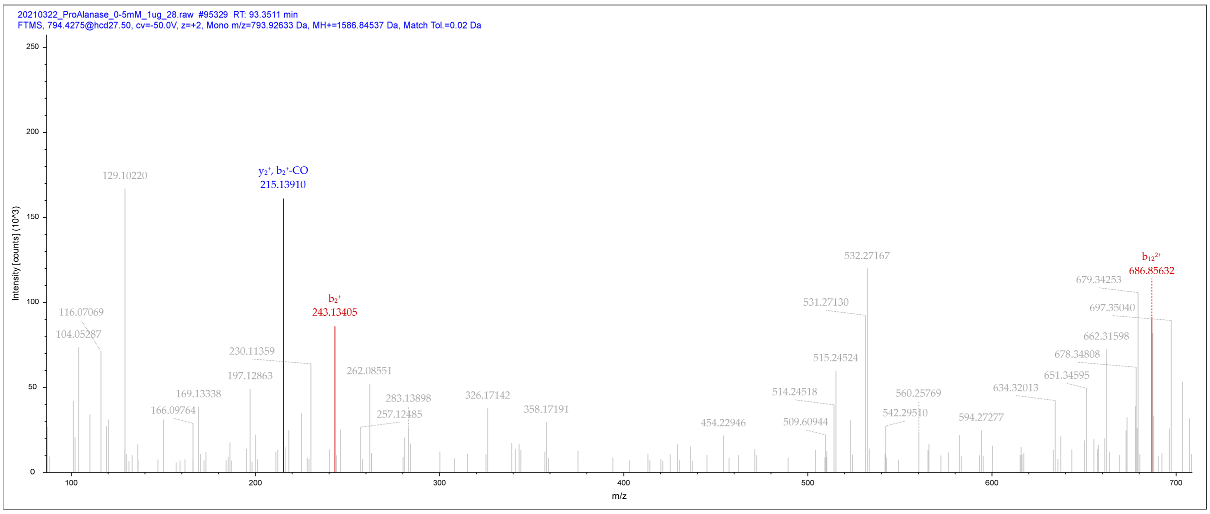

Glyceraldehyde-3-phosphate dehydrogenase (R263)

Sequence: VVKQASEGPLK, K3-Carboxyethyl (72.02113 Da)

Charge: +2, Monoisotopic m/z: 614.35129 Da (+0.49 mmu/+0.8 ppm), MH+: 1227.69531 Da, RT: 50.4006 min,

Identified with: Sequest HT (v1.17); **XCorr:2.39**, Percolator q-Value:2.6e-5, Percolator PEP:9.2e-5,

#1	b^+	b^{2+}	Seq.	y^+	y^{2+}	#2
1	100.07569	50.54148	V			11
2	199.14410	100.07569	V	1128.625...	564.81660	10
3	399.26020	200.13374	K-Carb...	1029.557...	515.28239	9
4	527.31877	264.16303	Q	829.44141	415.22434	8
5	598.35589	299.68158	A	701.38283	351.19505	7
6	685.38792	343.19760	S	630.34572	315.67650	6
7	814.43051	407.71889	E	543.31369	272.16048	5
8	871.45197	436.22962	G	414.27110	207.63919	4
9	968.50474	484.75601	P	357.24963	179.12845	3
10	1081.588...	541.29804	L	260.19687	130.60207	2
11			K	147.11280	74.06004	1

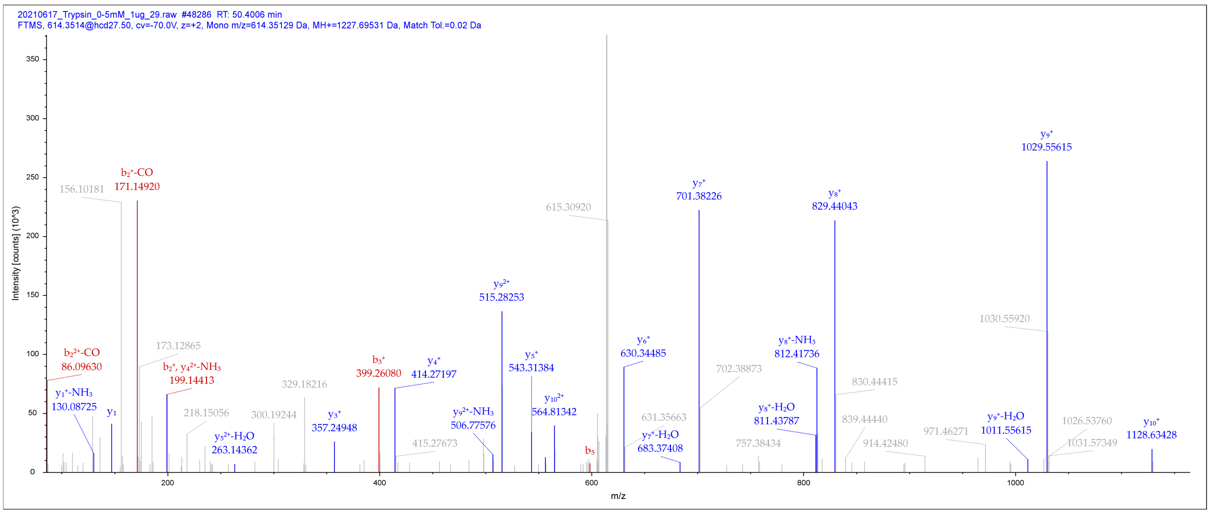

Glyceraldehyde-3-phosphate dehydrogenase (R215)

Sequence: GALQNIIPASTGAAKAVGK, K15-Carboxyethyl (72.02113 Da)

Charge: +3, Monoisotopic m/z: 613.68436 Da (+1.7 mmu/+2.77 ppm), MH+: 1839.03854 Da, RT: 88.4723 min,

Identified with: Sequest HT (v1.17); **XCorr:3.55**, Percolator q-Value:7.3e-6, Percolator PEP:1.0e-6,

#1	b⁺	b²⁺	b³⁺	Seq.	y⁺	y²⁺	y³⁺	#2
1	58.02874	29.51801	20.01443	G				19
2	129.06585	65.03657	43.69347	A	1782.011...	891.50963	594.67551	18
3	242.14992	121.57860	81.38816	L	1710.974...	855.99107	570.99647	17
4	370.20850	185.60789	124.07435	Q	1597.890...	799.44904	533.30178	16
5	484.25142	242.62935	162.08866	N	1469.832...	735.41975	490.61559	15
6	597.33549	299.17138	199.78335	I	1355.789...	678.39828	452.60128	14
7	710.41955	355.71341	237.47803	I	1242.705...	621.85625	414.90659	13
8	807.47231	404.23980	269.82896	P	1129.621...	565.31422	377.21191	12
9	878.50943	439.75835	293.50799	A	1032.568...	516.78784	344.86098	11
10	965.54146	483.27437	322.51867	S	961.53129	481.26928	321.18195	10
11	1066.589...	533.79821	356.20123	T	874.49926	437.75327	292.17127	9
12	1123.610...	562.30894	375.20838	G	773.45158	387.22943	258.48871	8
13	1194.647...	597.82749	398.88742	A	716.43012	358.71870	239.48156	7
14	1265.684...	633.34605	422.56646	A	645.39300	323.20014	215.80252	6
15	1465.800...	733.40410	489.27182	K-Carb...	574.35589	287.68158	192.12348	5
16	1536.838...	768.92265	512.95086	A	374.23980	187.62354	125.41812	4
17	1635.906...	818.45686	545.97367	V	303.20268	152.10498	101.73908	3
18	1692.927...	846.96759	564.98082	G	204.13427	102.57077	68.71627	2
19				K	147.11280	74.06004	49.70912	1

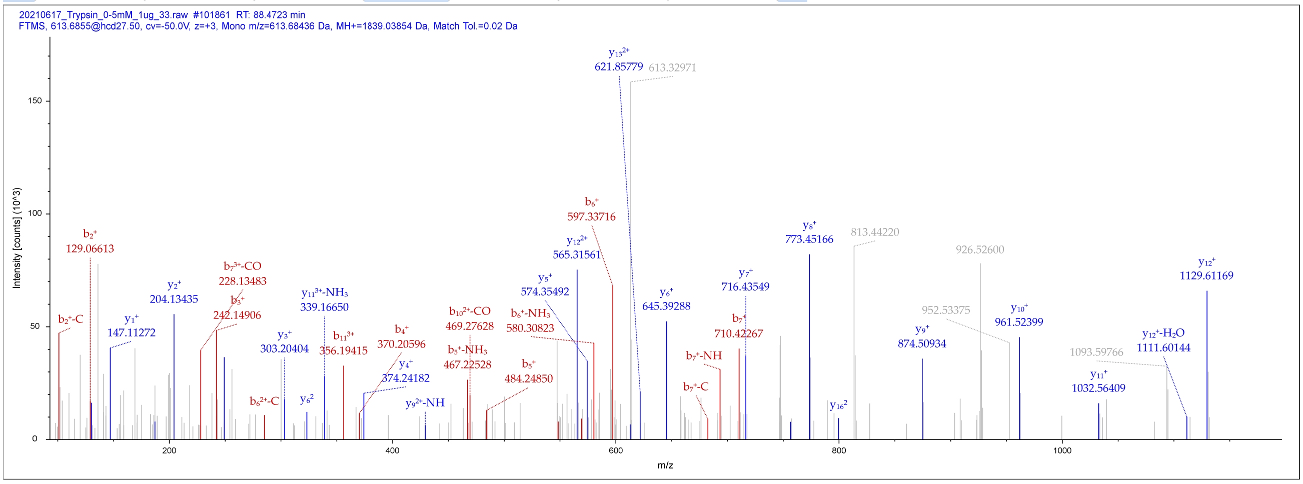

Pyruvate Kinase (K305)

Sequence: EKVFLAQKMMIGRCNRAGKP, C14-Carbamidomethyl (57.02146 Da), K2-Carboxyethyl (72.02113 Da)

Charge: +4, Monoisotopic m/z: 602.32367 Da (+4.05 mmu/+6.73 ppm), MH+: 2406.27286 Da, RT: 58.2619 min,

Identified with: Sequest HT (v1.17); **XCorr:3.52**, Percolator q-Value:5.9e-4, Percolator PEP:1.3e-3,

#1	b^+	b^{2+}	b^{3+}	b^{4+}	Seq.	y^+	y^{2+}	y^{3+}	y^{4+}	#2
1	130.04987	65.52857	44.02147	33.26792	E					20
2	330.16596	165.58662	110.72684	83.29695	K-Carb...	2277.214...	1139.110...	759.74288	570.05898	19
3	429.23438	215.12083	143.74964	108.06405	V	2077.097...	1039.052...	693.03751	520.02995	18
4	576.30279	288.65503	192.77245	144.83115	F	1978.029...	989.51842	660.01471	495.26285	17
5	689.38685	345.19706	230.46714	173.10217	L	1830.961...	915.98422	610.99190	458.49575	16
6	760.42397	380.71562	254.14617	190.86145	A	1717.877...	859.44219	573.29722	430.22473	15
7	888.48254	444.74491	296.83237	222.87609	Q	1646.839...	823.92363	549.61818	412.46545	14
8	1016.577...	508.79239	339.53069	254.89983	K	1518.781...	759.89434	506.93199	380.45081	13
9	1147.617...	574.31263	383.21085	287.65996	M	1390.686...	695.84686	464.23366	348.42707	12
10	1278.658...	639.83288	426.89101	320.42008	M	1259.645...	630.32662	420.55350	315.66695	11
11	1391.742...	696.37491	464.58570	348.69109	I	1128.605...	564.80637	376.87334	282.90683	10
12	1448.764...	724.88564	483.59285	362.94646	G	1015.521...	508.26434	339.17865	254.63581	9
13	1604.865...	802.93620	535.62656	401.97174	R	958.49994	479.75361	320.17150	240.38044	8
14	1764.895...	882.95152	588.97011	441.97940	C-Car...	802.39883	401.70305	268.13780	201.35517	7
15	1878.938...	939.97298	626.98441	470.49013	N	642.36818	321.68773	214.79425	161.34750	6
16	2035.039...	1018.023...	679.01812	509.51541	R	528.32526	264.66627	176.77994	132.83677	5
17	2106.076...	1053.542...	702.69716	527.27469	A	372.22415	186.61571	124.74623	93.81149	4
18	2163.098...	1082.052...	721.70431	541.53005	G	301.18703	151.09715	101.06720	76.05222	3
19	2291.193...	1146.100...	764.40263	573.55379	K	244.16557	122.58642	82.06004	61.79685	2
20					P	116.07061	58.53894	39.36172	29.77311	1

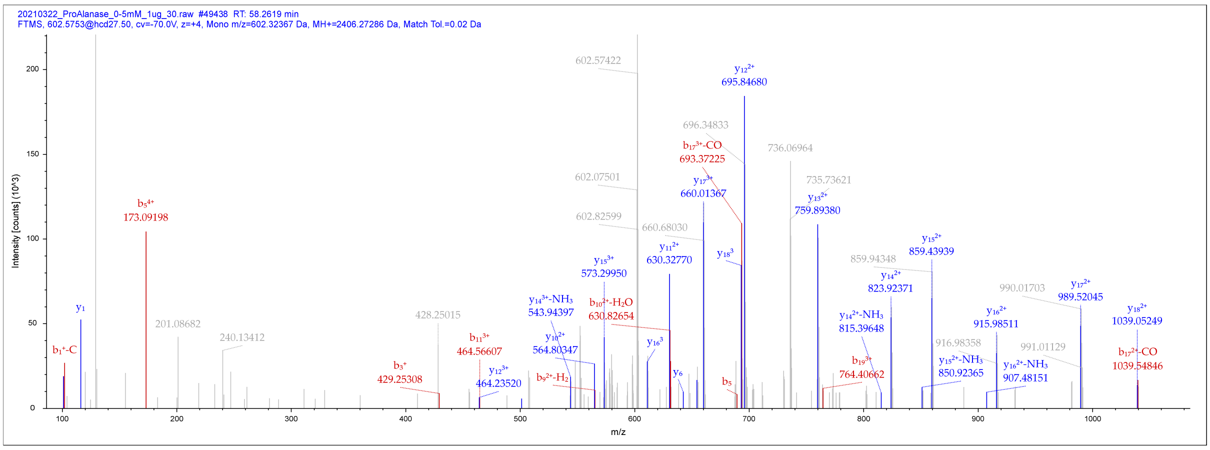

Pyruvate Kinase (R376)

Sequence: VRMQHLIAREA, M3-Oxidation (15.99492 Da), R2-MG-H1 (54.01057 Da)

Charge: +3,   Monoisotopic m/z: 465.25083 Da (+0.35 mmu/+0.75 ppm),   MH+: 1393.73793 Da,   RT: 59.8874 min,

Identified with: Sequest HT (v1.17); **XCorr:1.93**, Percolator q-Value:2.0e-3, Percolator PEP:8.1e-3,

#1	b^+	b^{2+}	b^{3+}	Seq.	y^+	y^{2+}	y^{3+}	#2
1	100.07569	50.54148	34.03008	V				11
2	310.18737	155.59732	104.06731	R-MG-...	1294.668...	647.83787	432.22767	10
3	457.22277	229.11502	153.07911	M-Oxi...	1084.556...	542.78203	362.19045	9
4	585.28134	293.14431	195.76530	Q	937.52139	469.26433	313.17865	8
5	722.34026	361.67377	241.45160	H	809.46281	405.23504	270.49246	7
6	835.42432	418.21580	279.14629	L	672.40390	336.70559	224.80615	6
7	948.50838	474.75783	316.84098	I	559.31984	280.16356	187.11146	5
8	1019.545...	510.27639	340.52002	A	446.23577	223.62152	149.41678	4
9	1175.646...	588.32694	392.55372	R	375.19866	188.10297	125.73774	3
10	1304.689...	652.84824	435.56792	E	219.09755	110.05241	73.70403	2
11				A	90.05496	45.53112	30.68984	1

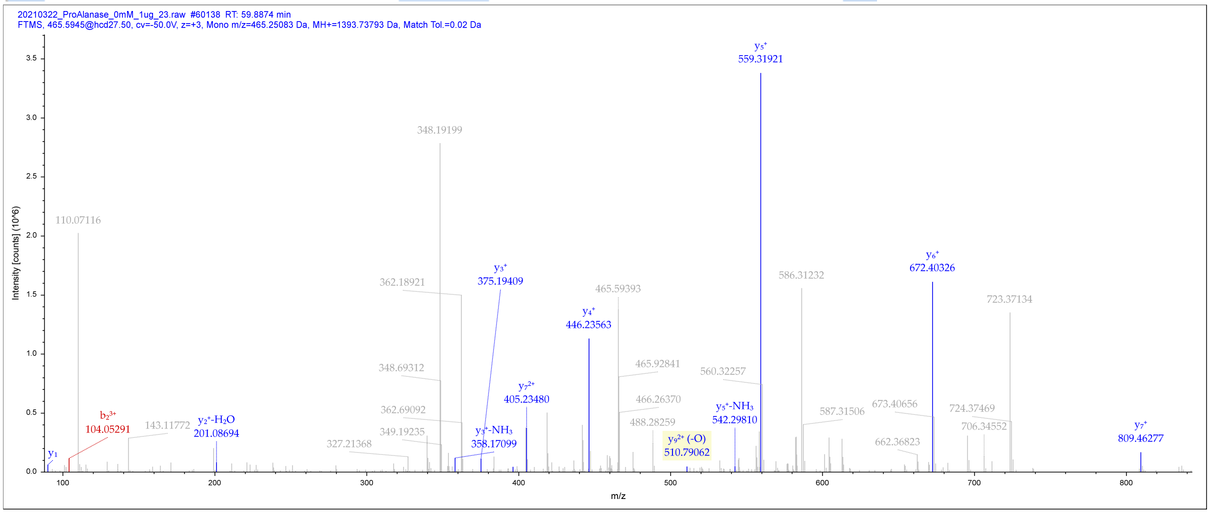

Pyruvate Kinase (R516)

Sequence: KGDVVIVLTGWRPGSGFTNTMR, R12-Carboxyethyl (72.02113 Da)

Charge: +3, Monoisotopic m/z: 821.76897 Da (+3.69 mmu/+4.5 ppm), MH+: 2463.29236 Da, RT: 94.1338 min,

Identified with: Sequest HT (v1.17); **XCorr:3.77**, Percolator q-Value:2.1e-5, Percolator PEP:8.0e-5,

#1	b^+	b^{2+}	b^{3+}	Seq.	y^+	y^{2+}	y^{3+}	#2
1	129.10224	65.05476	43.70560	K				22
2	186.12370	93.56549	62.71275	G	2335.186…	1168.096…	779.06696	21
3	301.15065	151.07896	101.05507	D	2278.164…	1139.586…	760.05981	20
4	400.21906	200.61317	134.07787	V	2163.137…	1082.072…	721.71749	19
5	499.28747	250.14738	167.10068	V	2064.069…	1032.538…	688.69469	18
6	612.37154	306.68941	204.79536	I	1965.001…	983.00418	655.67188	17
7	711.43995	356.22361	237.81817	V	1851.917…	926.46215	617.97719	16
8	824.52402	412.76565	275.51286	L	1752.848…	876.92794	584.95439	15
9	925.57169	463.28949	309.19542	T	1639.764…	820.38591	547.25970	14
10	982.59316	491.80022	328.20257	G	1538.716…	769.86207	513.57714	13
11	1168.672…	584.83987	390.22901	W	1481.695…	741.35134	494.56999	12
12	1396.794…	698.90099	466.26975	R-Car…	1295.616…	648.31169	432.54355	11
13	1493.847…	747.42738	498.62068	P	1067.493…	534.25057	356.50280	10
14	1550.868…	775.93811	517.62783	G	970.44109	485.72418	324.15188	9
15	1637.900…	819.45412	546.63851	S	913.41963	457.21345	305.14473	8
16	1694.922…	847.96485	565.64566	G	826.38760	413.69744	276.13405	7
17	1841.990…	921.49906	614.66847	F	769.36613	385.18671	257.12690	6
18	1943.038…	972.02290	648.35103	T	622.29772	311.65250	208.10409	5
19	2057.081…	1029.044…	686.36533	N	521.25004	261.12866	174.42153	4
20	2158.129…	1079.568…	720.04789	T	407.20712	204.10720	136.40722	3
21	2289.169…	1145.088…	763.72806	M	306.15944	153.58336	102.72466	2
22				R	175.11895	88.06311	59.04450	1

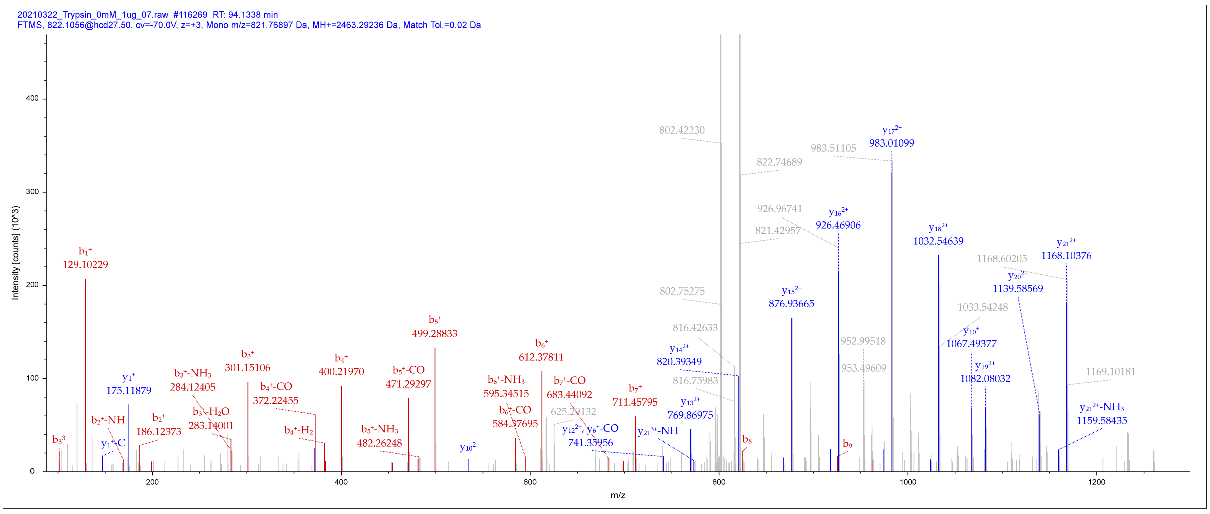

Triosephosphate isomerase (R5)

Sequence: PSRKFFVGGNWKMNGRKQSLGELIGTLNAAKVP, M13-Oxidation (15.99492 Da), R3-Carboxyethyl (72.02113 Da)

Charge: +5, Monoisotopic m/z: 738.60394 Da (+3.24 mmu/+4.38 ppm), MH+: 3688.99059 Da, RT: 93.3215 min,

Identified with: Sequest HT (v1.17); **XCorr:4.54**, Percolator q-Value:8.0e-5, Percolator PEP:1.6e-4,

#1	b^{+}	b^{2+}	b^{3+}	Seq.	y^{+}	y^{2+}	y^{3+}	#2
1	98.06004	49.53366	33.35820	P				33
2	185.09207	93.04967	62.36887	S	3591.921...	1796.464...	1197.978...	32
3	413.21431	207.11079	138.40962	R-Car...	3504.889...	1752.948...	1168.968...	31
4	541.30927	271.15827	181.10794	K	3276.767...	1638.887...	1092.927...	30
5	688.37769	344.69248	230.13075	F	3148.672...	1574.839...	1050.229...	29
6	835.44610	418.22669	279.15355	F	3001.604...	1501.305...	1001.206...	28
7	934.51451	467.76089	312.17636	V	2854.535...	1427.771...	952.18339	27
8	991.53598	496.27163	331.18351	G	2755.467...	1378.237...	919.16058	26
9	1048.557...	524.78236	350.19066	G	2698.445...	1349.726...	900.15343	25
10	1162.600...	581.80382	388.20497	N	2641.424...	1321.215...	881.14627	24
11	1348.679...	674.84348	450.23141	W	2527.381...	1264.194...	843.13196	23
12	1476.774...	738.89096	492.92973	K	2341.302...	1171.154...	781.10553	22
13	1623.810...	812.40866	541.94153	M-Oxi...	2213.207...	1107.107...	738.40720	21
14	1737.852...	869.43012	579.95584	N	2066.171...	1033.589...	689.39541	20
15	1794.874...	897.94086	598.96300	G	1952.128...	976.56801	651.38110	19
16	1950.975...	975.99141	650.99670	R	1895.107...	948.05727	632.37394	18
17	2079.070...	1040.038...	693.69502	K	1739.006...	870.00672	580.34024	17
18	2207.129...	1104.068...	736.38121	Q	1610.911...	805.95924	537.64192	16
19	2294.161...	1147.584...	765.39189	S	1482.852...	741.92995	494.95572	15
20	2407.245...	1204.126...	803.08658	L	1395.820...	698.41393	465.94505	14
21	2464.266...	1232.636...	822.09373	G	1282.736...	641.87190	428.25036	13
22	2593.309...	1297.158...	865.10793	E	1225.715...	613.36117	409.24321	12
23	2706.393...	1353.700...	902.80262	L	1096.672...	548.83987	366.22901	11
24	2819.477...	1410.242...	940.49731	I	983.58841	492.29784	328.53432	10
25	2876.498...	1438.753...	959.50446	G	870.50434	435.75581	290.83963	9
26	2977.546...	1489.276...	993.18702	T	813.48288	407.24508	271.83248	8
27	3090.630...	1545.818...	1030.881...	L	712.43520	356.72124	238.14992	7
28	3204.673...	1602.840...	1068.896...	N	599.35114	300.17921	200.45523	6
29	3275.710...	1638.358...	1092.575...	A	485.30821	243.15774	162.44092	5
30	3346.747...	1673.877...	1116.254...	A	414.27110	207.63919	138.76188	4
31	3474.842...	1737.924...	1158.952...	K	343.23398	172.12063	115.08285	3
32	3573.911...	1787.459...	1191.975...	V	215.13902	108.07315	72.38452	2
33				P	116.07061	58.53894	39.36172	1

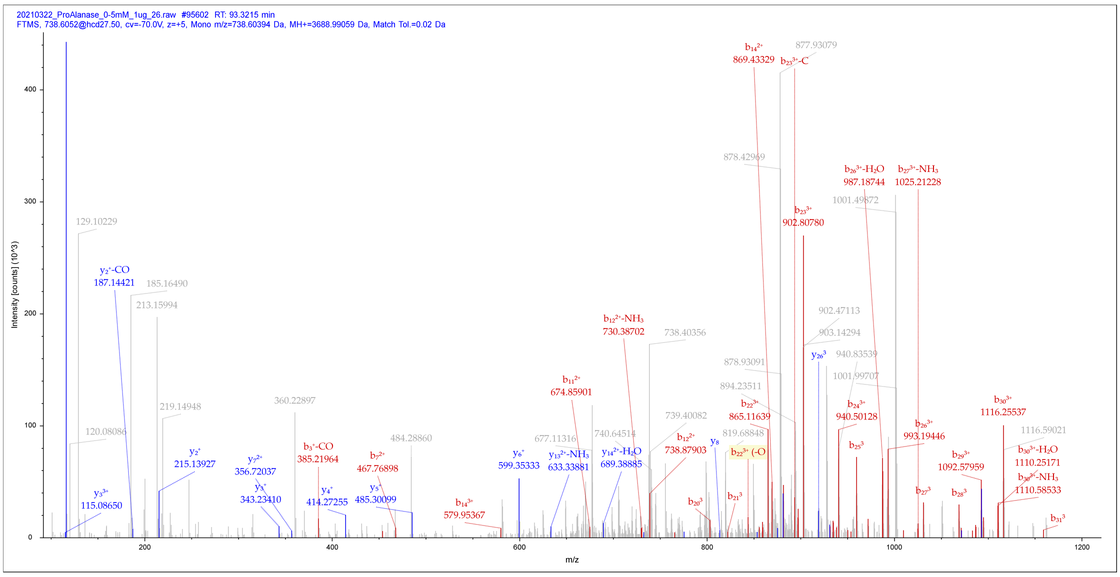

Glucose-6-phosphate isomerase (R370)

Sequence: SGTRVDHQTGPIVWGEPGTNGQHAFYQLIHQGTK, R4-Carboxyethyl (72.02113 Da)

Charge: +5, Monoisotopic m/z: 758.57929 Da (+2.92 mmu/+3.84 ppm), MH+: 3788.86736 Da, RT: 82.9100 min,

Identified with: Sequest HT (v1.17); **XCorr:2.26**, Percolator q-Value:2.5e-4, Percolator PEP:1.9e-3,

#1	b^+	b^{2+}	b^{3+}	Seq.	y^+	y^{2+}	y^{3+}	#2
1	88.03930	44.52329	30.01795	S				34
2	145.06077	73.03402	49.02511	G	3701.820...	1851.414...	1234.611...	33
3	246.10845	123.55786	82.70767	T	3644.799...	1822.903...	1215.604...	32
4	474.23069	237.61898	158.74841	R-Car...	3543.751...	1772.379...	1181.922...	31
5	573.29910	287.15319	191.77122	V	3315.629...	1658.318...	1105.881...	30
6	688.32604	344.66666	230.11353	D	3216.560...	1608.784...	1072.858...	29
7	825.38496	413.19612	275.79984	H	3101.534...	1551.270...	1034.516...	28
8	953.44353	477.22540	318.48603	Q	2964.475...	1482.741...	988.82989	27
9	1054.491...	527.74924	352.16859	T	2836.416...	1418.711...	946.14370	26
10	1111.512...	556.25998	371.17574	G	2735.368...	1368.188...	912.46114	25
11	1208.565...	604.78636	403.52666	P	2678.347...	1339.677...	893.45398	24
12	1321.649...	661.32839	441.22135	I	2581.294...	1291.150...	861.10306	23
13	1420.717...	710.86260	474.24416	V	2468.210...	1234.608...	823.40837	22
14	1606.797...	803.90225	536.27059	W	2369.142...	1185.074...	790.38557	21
15	1663.818...	832.41299	555.27775	G	2183.062...	1092.035...	728.35913	20
16	1792.861...	896.93428	598.29195	E	2126.041...	1063.524...	709.35198	19
17	1889.914...	945.46066	630.64287	P	1996.998...	999.00303	666.33778	18
18	1946.935...	973.97140	649.65002	G	1899.946...	950.47665	633.98686	17
19	2047.983...	1024.495...	683.33258	T	1842.924...	921.96592	614.97970	16
20	2162.026...	1081.516...	721.34689	N	1741.876...	871.44208	581.29714	15
21	2219.047...	1110.027...	740.35405	G	1627.833...	814.42061	543.28283	14
22	2347.106...	1174.056...	783.04024	Q	1570.812...	785.90988	524.27568	13
23	2484.165...	1242.586...	828.72654	H	1442.753...	721.88059	481.58949	12
24	2555.202...	1278.104...	852.40558	A	1305.695...	653.35114	435.90318	11
25	2702.270...	1351.638...	901.42838	F	1234.657...	617.83258	412.22415	10
26	2865.333...	1433.170...	955.78283	Y	1087.589...	544.29837	363.20134	9
27	2993.392...	1497.199...	998.46902	Q	924.52614	462.76671	308.84690	8
28	3106.476...	1553.741...	1036.163...	L	796.46756	398.73742	266.16071	7
29	3219.560...	1610.283...	1073.858...	I	683.38350	342.19539	228.46602	6
30	3356.619...	1678.813...	1119.544...	H	570.29944	285.65336	190.77133	5
31	3484.678...	1742.842...	1162.230...	Q	433.24052	217.12390	145.08503	4
32	3541.699...	1771.353...	1181.238...	G	305.18195	153.09461	102.39883	3
33	3642.747...	1821.877...	1214.920...	T	248.16048	124.58388	83.39168	2
34				K	147.11280	74.06004	49.70912	1

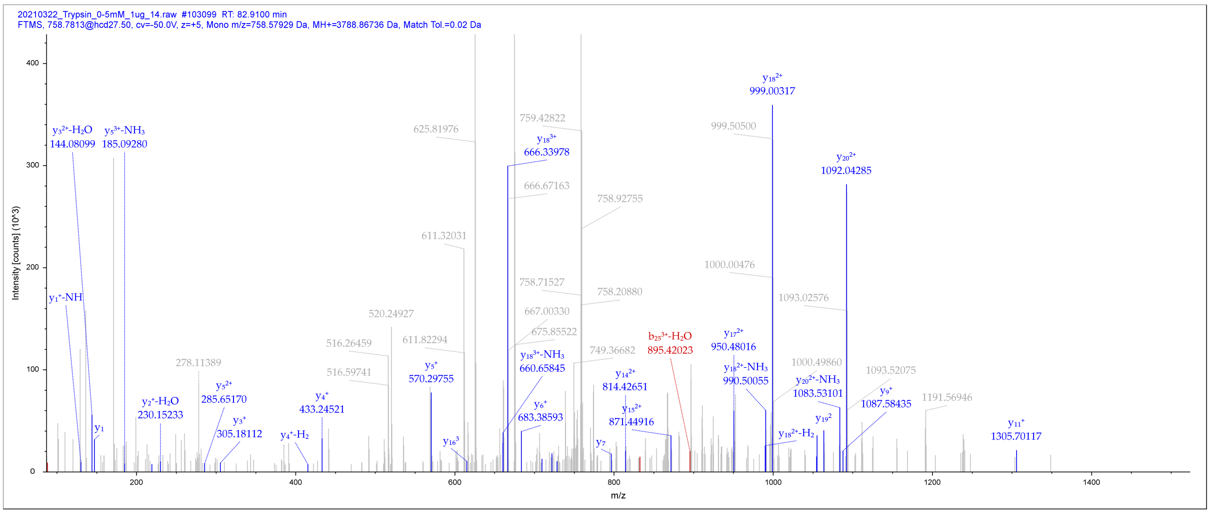

Glucose-6-phosphate isomerase (R6)

Sequence: MAALTRDPQFQK, R6-Carboxyethyl (72.02113 Da), M1-Met-loss+Acetyl (-89.02992 Da)

Charge: +2, Monoisotopic m/z: 694.86454 Da (+2.48 mmu/+3.57 ppm), MH+: 1388.72181 Da, RT: 68.9469 min,

Identified with: Sequest HT (v1.17); **XCorr:2.02**, Percolator q-Value:6.1e-4, Percolator PEP:5.3e-3,

#1	b⁺	b²⁺	Seq.	y⁺	y²⁺	#2
1	43.01784	22.01256	M-Met-...			12
2	114.05495	57.53112	A	1346.706...	673.85678	11
3	185.09207	93.04967	A	1275.669...	638.33823	10
4	298.17613	149.59170	L	1204.632...	602.81967	9
5	399.22381	200.11554	T	1091.548...	546.27764	8
6	627.34605	314.17666	R-Car...	990.50032	495.75380	7
7	742.37299	371.69014	D	762.37808	381.69268	6
8	839.42576	420.21652	P	647.35114	324.17921	5
9	967.48434	484.24581	Q	550.29837	275.65282	4
10	1114.552...	557.78001	F	422.23980	211.62354	3
11	1242.611...	621.80930	Q	275.17138	138.08933	2
12			K	147.11280	74.06004	1

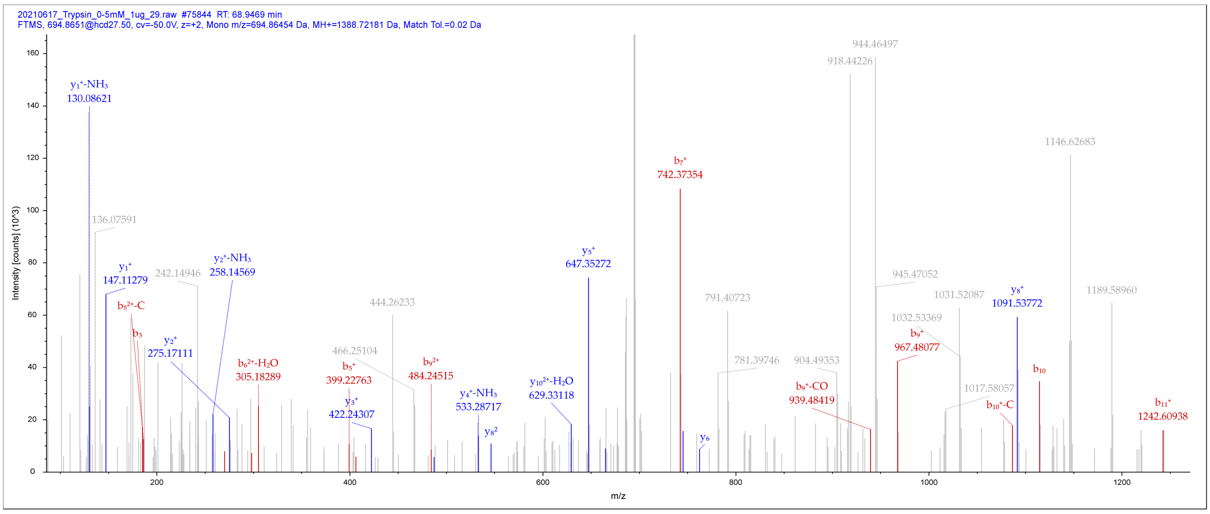

Supplement: Supplementary file 1 [file ijms-23-03689-s001.zip › ijms-1621919-Supplementary/Supplementary S3. MS2 spectra of MGO modified glycolytic enzymes.pdf]
